# Impact of SPP1 and HMOX1 Genes in Glioma: Correlations With Oncolytic Virus Infection, Adverse Prognosis and Increased Cell Proliferation

**DOI:** 10.1111/jcmm.70651

**Published:** 2025-06-11

**Authors:** Chunze Cui, Chunyan Wu, Shaoqi Zhang, Xiaofeng Yin

**Affiliations:** ^1^ Department of Neurosurgery Second Hospital of Shanxi Medical University Taiyuan China; ^2^ Operating Room Second Hospital of Shanxi Medical University Taiyuan China

**Keywords:** apoptosis, cancer therapy, glioma, oncolytic virus, prognosis

## Abstract

A high death rate among glioma patients is primarily due to poor prognostic outcomes and tumour metastasis. Oncolytic viruses have gained attention as a potential therapeutic strategy as eliminating tumour cells and modifying tumour microenvironment. This research highlights the urgent necessity to investigate novel therapeutic targets and clarify molecular mechanisms in glioma. The GSE166914 dataset was analysed to examine the SPP1 and HMOX1 expression after VSV‐M51 infection in glioma. By utilising the CancerSEA database, we assessed the potential function of SPP1/HMOX1 among pan‐cancer. Analysis of gene/protein expression levels and clinical significance was performed to identify the roles of SPP1/HMOX1 using TCGA‐glioma data. A correlation analysis was performed to screen co‐expressed genes, followed by GSEA analysis. qPCR and HPA analysis were utilised to assess the mRNA/protein levels of SPP1 and HMOX1 in glioma tissues. The anti‐apoptotic activity of SPP1 and HMOX1 was confirmed utilising the CCK‐8 assay and flow cytometry. VSV‐M51 infection resulted in SPP1/HMOX1 downregulation in T98 cells. The expression levels of SPP1/HMOX1 were significantly increased in glioma and associated with histological classifications and WHO grades. Elevated levels of SPP1/HMOX1 were related to poor prognosis in glioma. SPP1/HMOX1 was involved in influencing glioma cell motility through the PI3K/AKT, JAK–STAT and syndecan 1 signalling pathways. In vitro experiments showed higher expression levels of SPP1/HMOX1 in glioma tissues. Silencing SPP1/HMOX1 suppressed glioma cell proliferation and promoted apoptosis. In conclusion, dysregulated SPP1/HMOX1 expression was strongly related to glioma WHO grades and worse outcomes, providing deeper insights into glioma therapeutic targets and oncolytic virus‐based treatments.

## 1 Introduction

Glioma represents a common malignant form of brain cancer arising from the central nervous system (CNS) [1]. The aggressive characteristics of glioma result in poor overall survival for patients with glioma, which is less than 2 years [2, 3]. Although there have been significant advancements in the diagnosis and treatment of glioma, the prognosis for patients remains unfavourable, especially as the median survival of patients with glioblastoma (GBM) is 6–9 months [4, 5]. The main cause of death among glioma patients is the recurrence and metastasis of malignant cancer following surgical resection, chemotherapy and/or radiotherapy [3]. It emphasises the necessity of ongoing research into novel molecular targets to develop more effective and specific therapeutic options.

Oncolytic virus (OV) immunotherapy is categorised into naturally occurring viruses and genetically engineered viral agents [6, 7]. Oncolytic virotherapy involves the use of viruses, either in their natural state or genetically engineered, to selectively target and lyse cancer cells while protecting normal tissue [7, 8]. OV can be genetically modified to deliver therapeutic transgenes that act through four distinct mechanisms: (1) inducing oncolysis, (2) triggering vascular collapse, (3) stimulating anti‐tumour immune responses, and (4) producing therapeutic transgenes to combat malignancies [6, 9]. Several OVs are presently in clinical trials, with HSV‐1‐based OV representing a significant field of research [10]. Among these, T‐VEC stands out as the most clinically advanced, demonstrating potent cytotoxic effects among various cancer types, including melanoma and breast cancer [11, 12, 13]. Vesicular stomatitis virus (VSV)‐M51 infects cancer cells through a mechanism involving the activation of CD169 macrophages and dendritic cells, thereby inducing both oncolytic activity and anti‐tumour immune responses [14]. However, the relationship between OVs and their efficacy in glioma treatment has not been well understood.

Secreted phosphoprotein 1 (SPP1) is a secreted glycoprotein that widely expresses in tumour tissues and participates in tumour biological functions, including tumour cell proliferation, cell invasion and drug resistance [15]. Prior studies have highlighted its correlation with poor patient prognosis in different tumours, such as hepatocellular carcinoma [16], cervical cancer [17] and pancreatic tumour [18]. It is now accepted that heme‐oxygenase 1 (HMOX1), a stress response gene, regulates tumour cell ferroptosis [19]. A growing body of evidence shows that HMOX1 is involved in chemicals‐induced ferroptosis in liver cancer [20], gallbladder cancer [21] and ovarian cancer [22]. However, the roles of SPP1 and HMOX1 in glioma are not yet understood and require further exploration.

In this study, we investigated the regulatory mechanism of the VSV‐M51 in glioma pathogenesis. This study also examined the roles of SPP1 and HMOX1, alongside the in‐depth regulatory mechanisms in glioma. RNA‐sequencing analysis was performed utilising TCGA‐GBMLGG cohorts. Moreover, the co‐expressed genes with SPP1/HMOX1 were subjected to a GSEA analysis in order to explore regulatory signalling pathways activated by SPP1/HMOX1 in glioma. Overall, the findings demonstrated that SPP1/HMOX1 were associated with poor overall survival in glioma patients, indicating their potential as clinical prognostic and therapeutic targets of oncolytic virus treatment.

## 2 Materials and Methods

### 2.1 Analysis of GSE166914 Dataset

RNA‐seq counts of GSE166914 dataset were downloaded from the NCBI website (https://www.ncbi.nlm.nih.gov/geo/query/acc.cgi?acc=GSE166914). GSE166914 dataset was submitted by Jingshu X, Sun Yat‐sen University, Shenzhen, China. Human glioma T98 cells were infected with oncolytic virus VSV‐M51 for 24 h, collected using Trizol and subjected to high throughput sequencing on the GPL23227 BGISEQ‐500 platform. The three samples (GSM5087966, GSM5087967 and GSM5087968) are the DMSO‐treated cells. The GSM5087969, GSM5087970 and GSM5087971 datasets are collected from VSV‐M51‐treated T98 cells.

### 2.2 CancerSEA Analysis

To evaluate cancer cell function, we utilised the CancerSEA database to perform a single‐cell analysis. Further correlation analysis identified associations between the levels of SPP1/HMOX1 and several functional statuses.

### 2.3 Expression Levels of SPP1/HMOX1 and Clinical Significance in Glioma

We evaluated the expression levels of SPP1 and HMOX1 in both normal tissues and glioma samples using TCGA clinical datasets and the R package for analysis. For glioma samples, the data were sourced from the TCGA‐GBMLGG dataset, and normal tissue datasets were obtained from the GTEx project. Wilcoxon test and Kruskal‐Wallis test were carried out to analyse the statistical significance between normal and glioma tissues, and clinical parameters correlations.

### 2.4 Survival Analysis in Glioma

To evaluate the prognostic values of SPP1 and HMOX1 in patients with glioma, we performed Kaplan–Meier survival curves to assess overall survival (OS), disease‐free survival (DSS) and progression‐free interval (PFI). Two groups (high expression and low expression groups) were generated based on the median mRNA expression of SPP1and HMOX1. The univariate and multivariate analyses were also carried out to correlate between clinical risk factors and OS.

### 2.5 GSEA Analysis

GSEA analysis evaluated the enriched signalling pathways which might be regulated by SPP1/HMOX1 as an enrichment score (NES) > 1, *p*.adjust < 0.05 and *q* < 0.25.

### 2.6 RNA Extraction and RT‐qPCR Analysis in Glioma Tissues or Cells

This study enrolled 20 glioma patients who underwent surgery from 2021 to 2023. None of the 20 patients had received any preoperative treatments, such as radiotherapy, chemotherapy, targeted therapy or immunotherapy, to ensure that the tissue samples were not affected by treatment‐related changes. After surgical excision, glioma tissues and normal brain tissues were quickly stored in liquid nitrogen. The research received approval from the Ethics Committee of Second Hospital of Shanxi Medical University. Total RNAs were extracted from clinical tissues and glioma cell lines using TRIzol Reagent (Invitrogen). Subsequently, cDNA was synthesised from the extracted RNA using PrimeScript RT Master Mix (Takara, Japan). qPCR analysis was carried out utilising SYBR Green SuperMix (Bio‐Rad, USA) and employing Real‐Time PCR System (Applied Biosystems, USA). The quantification of SPP1 and HMOX1 expression levels was analysed using the 2 −ΔΔCt method, with GAPDH employed as the housekeeping gene for normalisation. The primers of SPP1/HMOX1 are listed as follows: SPP1 forward primer 5′‐AGG CAT CAC CTG TGC CAT AC‐3′, SPP1 reverse primer 5′‐GTC CAA GCT TCT GGG GAC AA‐3′, HMOX1 forward primer 5′‐GGG AAT TCT CTT GGC TGG CT‐3′, HMOX1 reverse primer 5′‐GCT GCC ACA TTA GGG TGT CT‐3′.

### 2.7 Cell Culture and Transfection

Glioma cell lines (U87MG and U251) were grown in a sterile environment with DMEM supplemented with 10% FBS (Life Technologies, USA) in an incubator maintained at 37°C with 5% CO_2_. siRNAs targeting SPP1/HMOX1 were transfected into glioma cells utilising Lipofectamine 3000. si‐SPP1#1: forward 5′‐UCU UUA GUG CUG CUU UUC CUC‐3′, reverse 5′‐GGA AAA GCA GCA CUA AAG AUG‐3′. si‐SPP1#2: forward 5′‐AUC UUU AGU GCU GCU UUU CCU‐3′, reverse 5′‐GAA AAG CAG CAC UAA AGA UGU‐3′. si‐HMOX1#1: forward 5′‐AGA AUC UUG CAC UUU GUU GCU‐3′, reverse 5′‐CAA CAA AGU GCA AGA UUC UGC‐3′. si‐HMOX1#2: forward 5′‐AUU CAC AUG GCA UAA AGC CCU‐3′, reverse 5′‐GGC UUU AUG CCA UGU GAA UGC‐3′.

### 2.8 Cell Viability Assessment

The viability of glioma cells transfected with siRNAs/siNCs was assessed by performing a Cell Counting Kit‐8 assay. A cell suspension was cultured in a 96‐well flat‐bottomed plate and incubated for 0, 24, 48 and 72 h in a cell incubator. 10 μL of CCK‐8 solution, obtained from Solarbio (Beijing), is added to each well of the microplate. The microplate is incubated for 3 h to allow the CCK‐8 reagent to interact with the cells. After incubation, the absorbance at 450 nm is measured using a microplate reader.

### 2.9 Apoptosis Quantification

Following siRNA transfection, the adherent cells were gathered, rinsed twice with cold PBS, and subjected to annexin V‐FITC and PI staining. To determine the apoptotic ratio, U87MG and U251 cells were treated with annexin V and PI using the FITC Annexin V Apoptosis Detection Kit (Invitrogen, USA) and evaluated using a flow cytometer (BD Bioscience, USA).

### 2.10 Statistics Analysis

All experiments were performed in at least three independent biological replicates. Statistical analyses, including one‐way and two‐way ANOVA, were conducted using GraphPad Prism (version 8). Results are expressed as mean ± standard deviation. A *p*‐value of less than 0.05 was considered as statistically significant.

## 3 Results

### 3.1 Differential Gene Analysis of VSV‐M51‐Infected T98 Cell Sequencing Dataset

**FIGURE 1:**
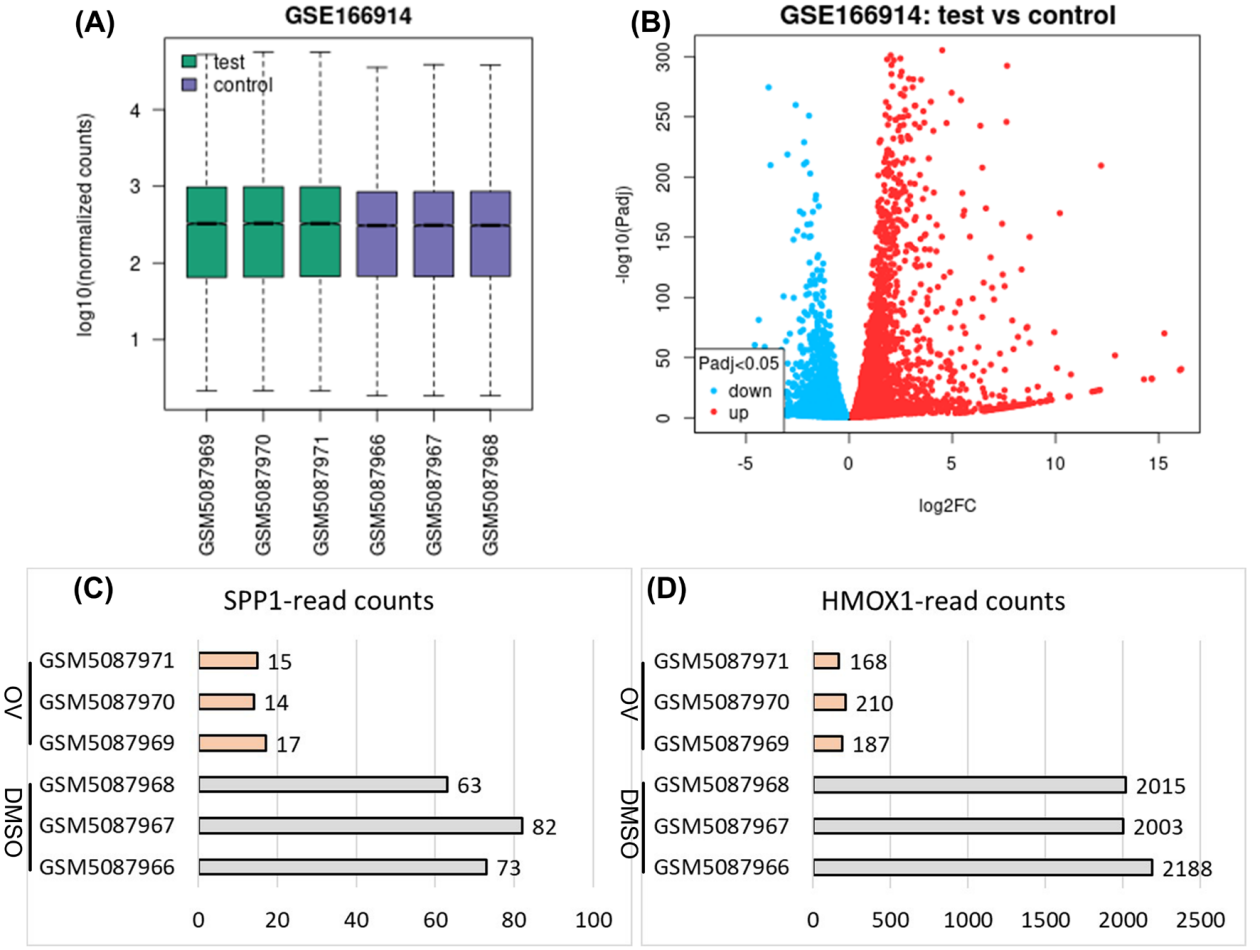
Differential gene expression analysis in VSV‐M51‐infected glioma cells. (A) The distribution of gene expression values across all samples is shown. (B) A volcano plot illustrates DEGs between VSV‐M51‐infected glioma cells and DMSO‐treated controls. (C, D) Expression levels of SPP1 and HMOX1 are presented for each GSM sample.

The GSE166914 dataset included gene expression profiles from glioma T98 cells exposed to VSV‐M51 or DMSO treatment. The selected samples were suitable for differential expression analysis by viewing the distribution of median‐centered values (Figure 1A). Figure 1B generated a volcano plot which highlighted DEGs with statistical significance (*p* < 0.05). In VSV‐M51‐exposed T98 cells, a total of 1756 DEGs exhibited markedly increased expression, while 1409 DEGs showed decreased expression (Figure 1B). Figure 1C,D demonstrated that the read counts of SPP1/HMOX1 were significantly lower in the VSV‐M51‐treated group in comparison with the DMSO‐treated group (all *p* < 0.001). These results demonstrated that SPP1 and HMOX1 might act as oncogenes in glioma that were inhibited by VSV‐M51 treatment.

### 3.2 Relevance of SPP1/HMOX1 Across Several Functional Status in Pan‐Cancer

**FIGURE 2:**
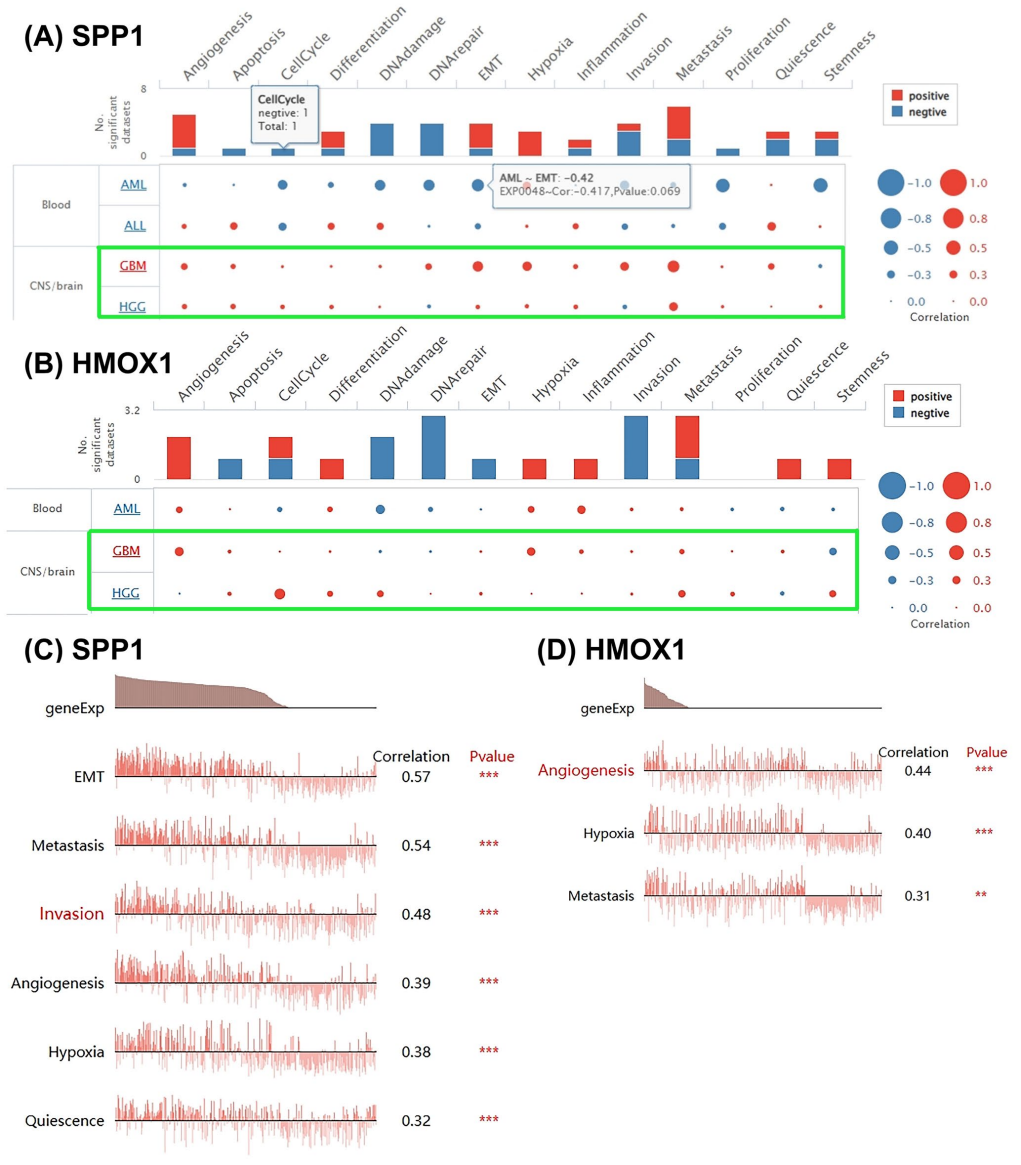
Expression patterns of SPP1 and HMOX1 across pan‐cancer genomes. CancerSEA analysis demonstrated a correlation between the expression levels of (A) (C) SPP1 and (B) (D) HMOX1 genes across different cancer types.

Our research represents the first systematic evaluation of SPP1 and HMOX1 functional signatures at the single‐cell level utilising the CancerSEA database. The results revealed that there are significant positive correlations between SPP1 and functional status in glioma, including EMT (correlation *R* = 0.57), metastasis (correlation *R* = 0.54) and invasion (correlation *R* = 0.48) (Figure 2A,C). Similarly, Figure 2B,D demonstrated that HMOX1 expression might be related to glioma angiogenesis (correlation *R* = 0.44), hypoxia (correlation *R* = 0.40) and metastasis (correlation *R* = 0.31). These findings highlighted a potential role of SPP1/HMOX1 in glioma progression.

### 3.3 Clinicopathological Characteristics of Glioma Patients and Correlation With SPP1/HMOX1 Expression

**FIGURE 3:**
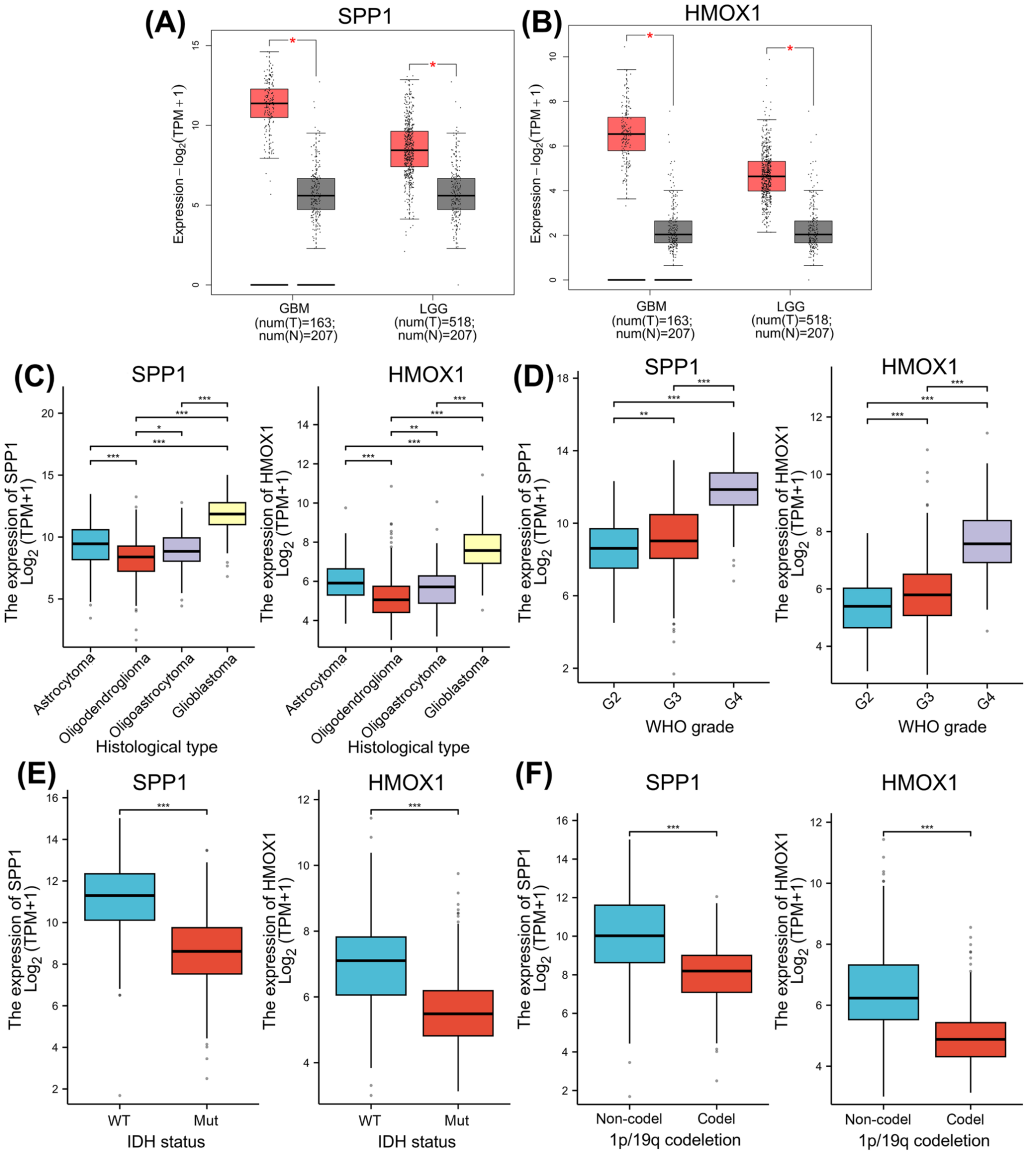
Elevated expression of SPP1 and HMOX1 in TCGA‐GBM/LGG datasets. The expression levels of (A) SPP1 and (B) HMOX1 in glioma datasets from TCGA and GTEx are shown. The box plots demonstrate mRNA expression levels of SPP1 and HMOX1 among different (C) histological types, (D) WHO grades, (E) IDH status and (F) 1p/19q codeletion in glioma. *p < 0.05, **p < 0.01, ***p < 0.001.

Next, we performed RNA‐sequencing analysis to analyse the clinical significance of SPP1/HMOX1 in a cohort of TCGA‐LGG/GBM samples. The GTEx samples served as the control group for *t*‐test comparison. Figure 3A,B indicated that the log2(TPM + 1) values of SPP1/HMOX1 in clinical GBM/LGG tissues (*n* = 163) were markedly increased compared to TCGA normal and GTEx samples (*n* = 207) (*p* < 0.01). In addition, the elevated SPP1 and HMOX1 expression levels were found to be associated with more advanced histological subtypes of glioma, specifically when comparing GBM to astrocytoma, oligodendroglioma and oligoastrocytoma (*p* < 0.001, Figure 3C). The significant elevated expression of SPP1 and HMOX1 was detected in the samples diagnosed with classified as WHO G4/G3 gliomas in comparison to those with G2 glioma tissues (*p* < 0.001, Figure 3D). This suggests a potential correlation between SPP1/HMOX1 expression and the degree of tumour malignancy. Furthermore, baseline data comprising clinicopathological characteristics of glioma patients are shown in Table 1. The data revealed that high SPP1/HMOX1 expression is positively related to patient age, IDH WT status, 1p/19q codeletion, and primary treatment outcome (Figure 3E,F), suggesting that SPP1 and HMOX1 play crucial roles in the progression of glioma (*p* < 0.001).

**TABLE 1:** Baseline clinical characteristics data of glioma patients.

Characteristics	SPP1			HMOX1		
	Low expression	High expression	*p*	Low expression	High expression	*p*
*n*	349	350		349	350	
Gender, *n* (%)						
Female	152 (21.7%)	146 (20.9%)	0.62	165 (23.6%)	133 (19%)	**0.01**
Male	197 (28.2%)	204 (29.2%)		184 (26.3%)	217 (31%)	
Age, *n* (%)						
< = 60	307 (43.9%)	249 (35.6%)	**3.53E‐08**	307 (43.9%)	249 (35.6%)	**3.5E‐08**
> 60	42 (6%)	101 (14.4%)		42 (6%)	101 (14.4%)	
WHO grade, *n* (%)						
G2	155 (24.3%)	69 (10.8%)	**6.04E‐34**	158 (24.8%)	66 (10.4%)	**1.49E‐38**
G3	138 (21.7%)	107 (16.8%)		137 (21.5%)	108 (17%)	
G4	14 (2.2%)	154 (24.2%)		9 (1.4%)	159 (25%)	
Histological type, *n* (%)						
Astrocytoma	96 (13.7%)	100 (14.3%)	**3.33E‐40**	98 (14%)	98 (14%)	**4.17E‐45**
Oligodendroglioma	156 (22.3%)	44 (6.3%)		158 (22.6%)	42 (6%)	
Oligoastrocytoma	83 (11.9%)	52 (7.4%)		84 (12%)	51 (7.3%)	
Glioblastoma	14 (2%)	154 (22%)		9 (1.3%)	159 (22.7%)	
IDH status, *n* (%)						
WT	42 (6.1%)	204 (29.6%)	**1.89E‐38**	53 (7.7%)	193 (28%)	**1.76E‐29**
Mut	304 (44.1%)	139 (20.2%)		294 (42.7%)	149 (21.6%)	
1p/19q codeletion, *n* (%)						
Non‐codel	207 (29.9%)	313 (45.2%)	**2.45E‐22**	202 (29.2%)	318 (46%)	**2.96E‐26**
Codel	142 (20.5%)	30 (4.3%)		147 (21.2%)	25 (3.6%)	
Primary therapy outcome, *n* (%)						
PD	56 (12%)	56 (12%)	**0.01**	62 (13.3%)	50 (10.8%)	0.13
SD	99 (21.3%)	49 (10.5%)		96 (20.6%)	52 (11.2%)	
PR	46 (9.9%)	19 (4.1%)		46 (9.9%)	19 (4.1%)	
CR	96 (20.6%)	44 (9.5%)		94 (20.2%)	46 (9.9%)	

*Note:* Bold *p* values presents as statistically significance.

### 3.4 High SPP1 and HMOX1 Expression Are Correlated With a Worse Prognosis in Glioma

**FIGURE 4:**
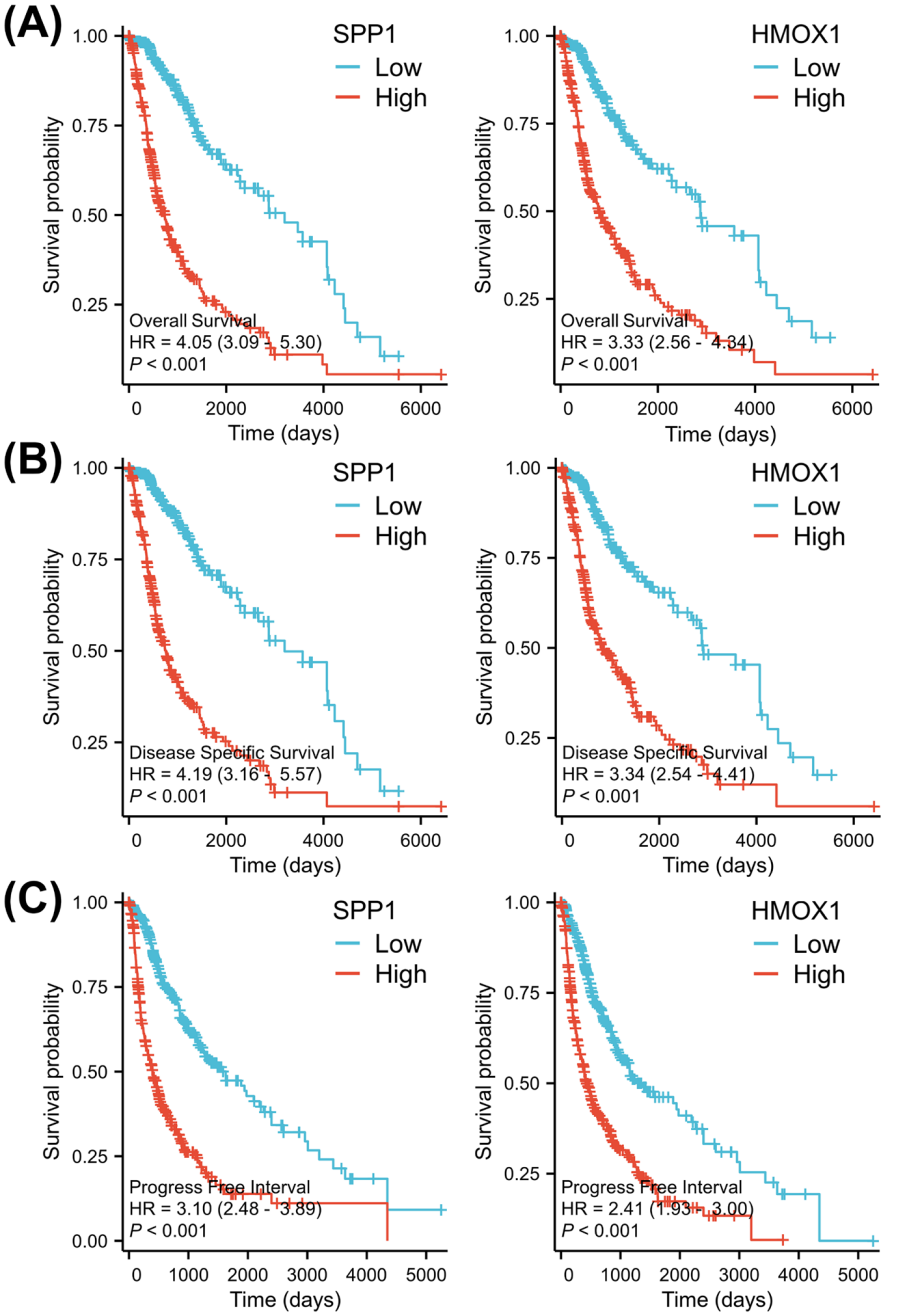
Prognostic significance of SPP1 and HMOX1 in glioma. The Kaplan–Meier method was used to assess the correlation between SPP1/HMOX1 expression levels and clinical outcomes, such as (A) OS, (B) DSS and (C) PFI, in patients with glioma who had high or low expression levels of SPP1/HMOX1.

To evaluate the prognostic significance of SPP1/HMOX1 overexpression in glioma specimens, we conducted Kaplan–Meier survival analysis. SPP1 overexpression, as confirmed by gene expression grouping analysis, is correlated with a significant decrease in survival probability, including OS, DSS and PFI, compared to the low gene expression group (*p* < 0.001, Figure 4). Glioma patients exhibiting high levels of HMOX1 expression had a significantly shorter OS compared to those with lower HMOX1 expression (*p* < 0.001, Figure 4). Figure S1 showed that patients with high‐expression scores, which were calculated for the SPP1 and HMOX1 signatures in LGG samples, had significantly poor survival rates (*p* < 0.05). Furthermore, univariate Cox regression analysis demonstrated that high SPP1 and HMOX1 expression (*p* < 0.001) emerged as independent prognostic factors, similar to WHO status, IDH status and 1p/19q codeletion (Table 2). These findings imply that SPP1/HMOX1, as identified biomarkers, hold significant clinical value in predicting unfavourable outcomes in glioma patients.

**TABLE 2:** Univariate and multivariate cox regression analysis.

Characteristics	Total (*N*)	Univariate analysis		Multivariate analysis	
		HR(95% CI)	*p*	HR(95% CI)	*p*
SPP1					
Low	348				0.184
High	350	4.049 (3.094–5.299)	**< 0.001**	1.333 (0.872–2.037)	
HMOX1					
Low	348				0.289
High	350	3.330 (2.557–4.338)	**< 0.001**	1.248 (0.829–1.879)	
Age					
< = 60	555				**< 0.001**
> 60	143	4.696 (3.620–6.093)	**< 0.001**	4.144 (2.512–6.835)	
Gender					
Female	297				**0.009**
Male	401	1.250 (0.979–1.595)	0.073	1.819 (1.165–2.841)	
WHO grade					
G2	223				
G3	245	2.967 (1.986–4.433)	**< 0.001**	1.970 (1.249–3.109)	**0.004**
G4	168	18.600 (12.448–27.794)	**< 0.001**	6.194 (1.972–19.453)	**0.002**
IDH status					
WT	246				**0.027**
Mut	442	0.116 (0.089–0.151)	**< 0.001**	0.549 (0.323–0.933)	
1p/19q codeletion					
Non‐codel	520				0.187
Codel	171	0.225 (0.147–0.346)	**< 0.001**	0.689 (0.396–1.199)	
Primary therapy outcome					
PD	112				
SD	148	0.440 (0.294–0.658)	**< 0.001**	0.374 (0.225–0.621)	**< 0.001**
PR	65	0.167 (0.073–0.385)	**< 0.001**	0.199 (0.071–0.557)	**0.002**
CR	139	0.131 (0.063–0.273)	**< 0.001**	0.166 (0.077–0.358)	**< 0.001**

*Note:* Bold *p* values presents as statistically significance.

### 3.5 Identification of 20 Key Proteins Related to SPP1/HMOX1 for Glioma via Constructing GSEA Analysis

**FIGURE 5:**
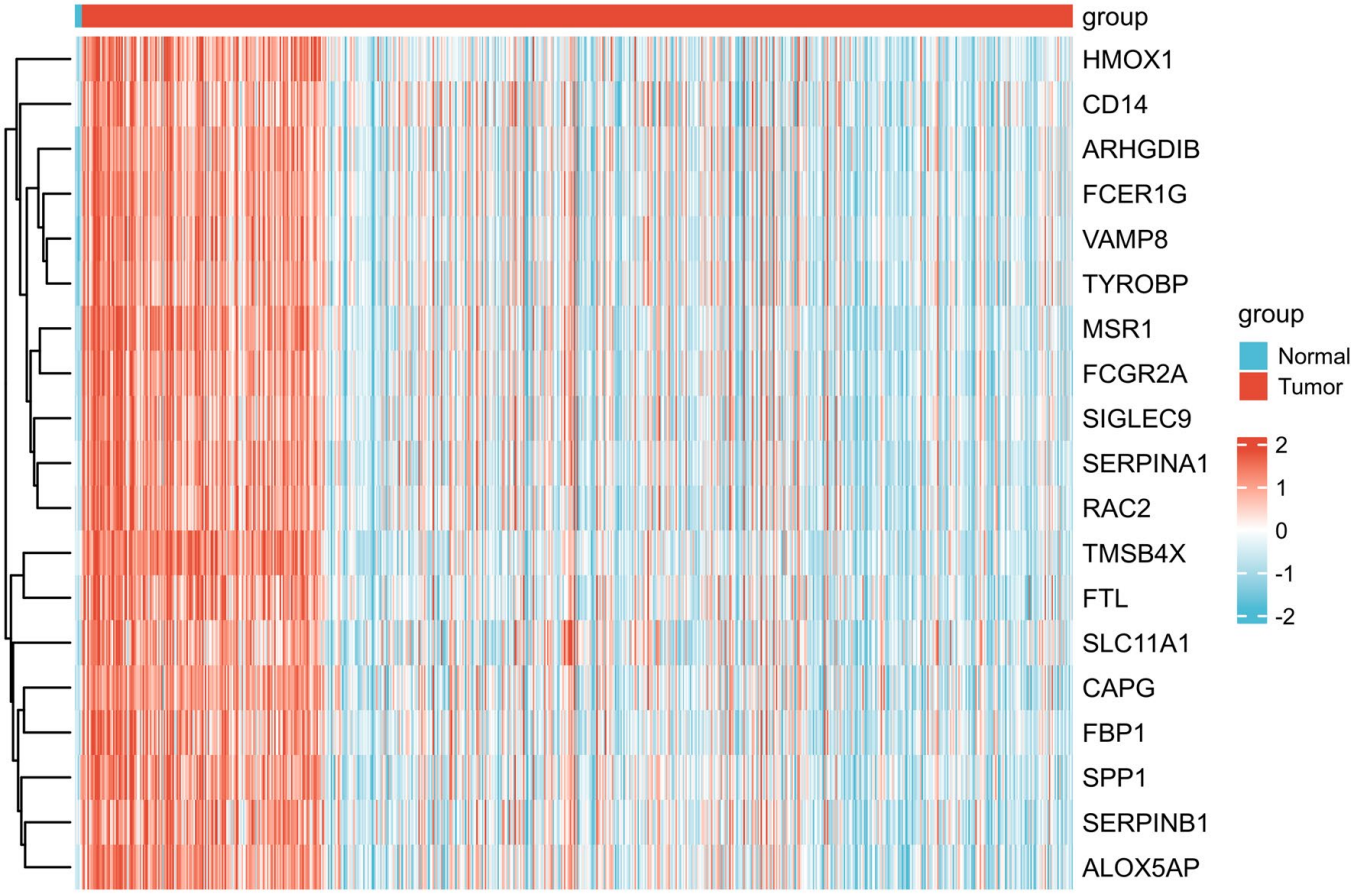
Analysis of SPP1/HMOX1‐Related genes in glioma. Heatmaps showing the top 20 highly expressed genes in gliomas with SPP1/HMOX1 expression.

To delve deeper into the mechanisms underlying gene interactions, we employed a single‐gene differential expression analysis to determine potential co‐expressed genes that strongly correlated with SPP1/HMOX1 in glioma. As a co‐expressed gene, several specific conditions must be satisfied: (A) Pearson's correlation coefficient |*R*| > = 0.75; (B) Pearman's correlation coefficient |*R*| > = 0.75; (C) *p* value less than 0.05. Each one of the two correlation coefficients manifested that both SPP1 and HMOX1 were consistently co‐expressed with 20 genes in glioma samples, such as VAMP8, RAC2, MSR1, CAPG and FBP1 (Figure 5).

**FIGURE 6:**
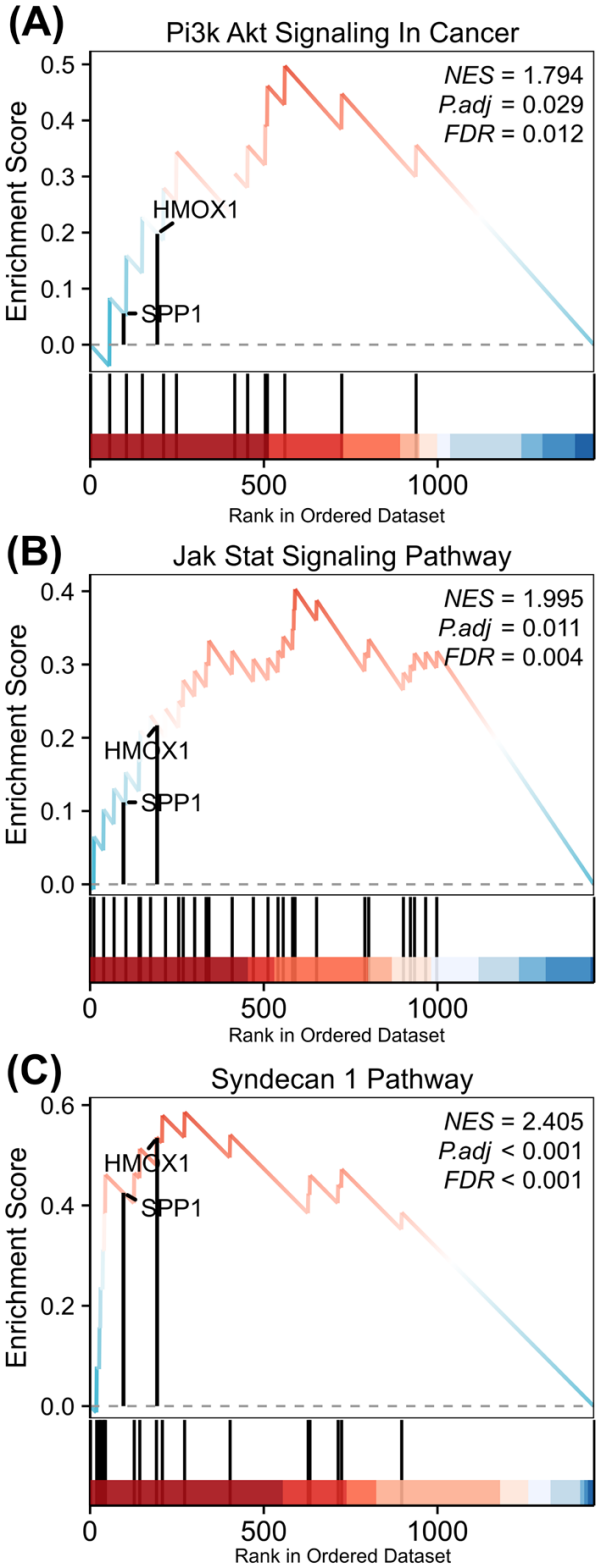
GSEA analysis in glioma. (A–C) GSEA reveals the activation of signalling pathways associated with elevated expression levels of SPP1 and HMOX1 in glioma.

To elucidate the molecular mechanism by which SPP1/HMOX1 contribute to glioma development, we utilised TCGA‐GBMLGG data cohorts to carry out GSEA analysis. Our findings demonstrated a significant activation of the PI3K‐AKT pathway (NES = 1.794, *p* = 0.029, FDR = 0.012) in the higher SPP1 expression group (Figure 6A). The PI3K‐AKT signalling pathway was identified in glioma, suggesting its role in gene mutations and the progression of glioma malignancy [23]. Furthermore, our findings revealed a hallmark of the JAK–STAT signalling pathway (NES = 1.995, *p* = 0.011, FDR = 0.004) and the syndecan 1 pathway in gliomas expressing HMOX1 (NES = 2.405, *p* < 0.001, FDR < 0.001) (Figure 6B,C). The results from GSEA enrichment indicated that elevated expression of SPP1/HMOX1 was strongly associated with glioma malignancy.

### 3.6 SPP1 and HMOX1 Are Highly Expressed in Glioma Tissues

**FIGURE 7:**
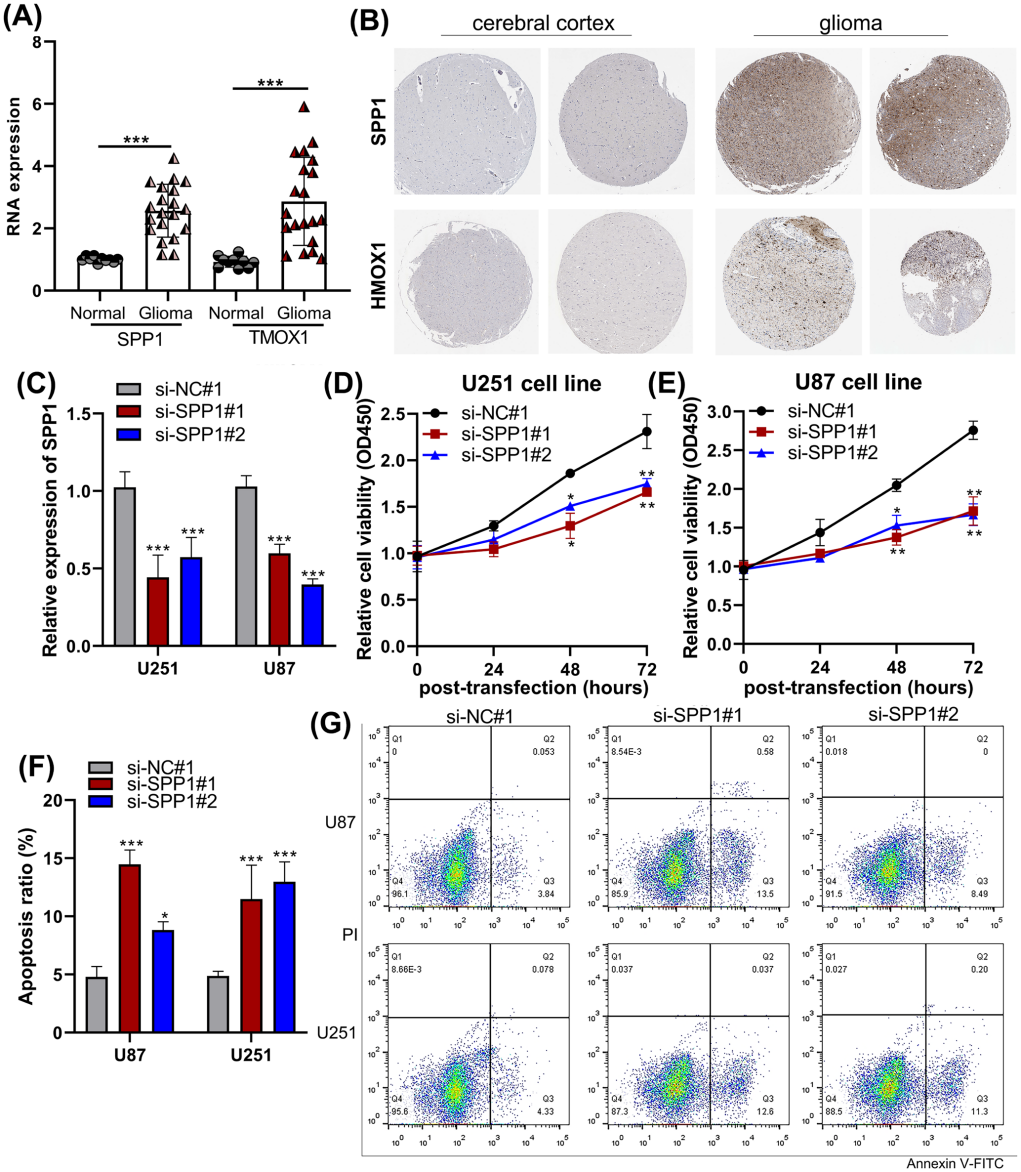
Silencing SPP1 reduces cell viability and increases apoptosis in glioma cells. (A) Quantitative PCR (qPCR) analysis of SPP1 and HMOX1 mRNA expression in glioma tissues and non‐tumour tissues. (B) Immunohistochemistry (IHC) analysis of SPP1 and HMOX1 protein expression levels in glioma tissues and cerebral cortex tissues. (C) Expression levels of SPP1 in si‐SPP1‐transfected cells, as measured by RT‐qPCR. (D, E) CCK‐8 assays were conducted to assess changes in cellular viability following SPP1 downregulation. (F, G) Flow cytometric analysis demonstrates alterations in the apoptotic rate of glioma cells transfected with si‐SPP1. *n* = 3. The data are presented as mean ± SD. *p < 0.05, **p < 0.01, ***p < 0.001.

To examine the role of SPP1 or HMOX1 in the malignant progression of glioma, a total of 20 clinical tissue samples were obtained from glioma patients. We subsequently evaluated the mRNA expression in a cohort of 20 glioma tissues using RT‐qPCR and found that SPP1 or HMOX1 was significantly overexpressed in glioma compared with non‐tumour tissues (*p* < 0.001, Figure 7A). Furthermore, immunohistochemical analysis showed that SPP1 and HMOX1 were significantly upregulated in glioma tissues compared to cerebral cortex tissues (Figure 7B).

### 3.7 Knockdown SPP1 and HMOX1 Promoted Apoptosis in Glioma Cells

**FIGURE 8:**
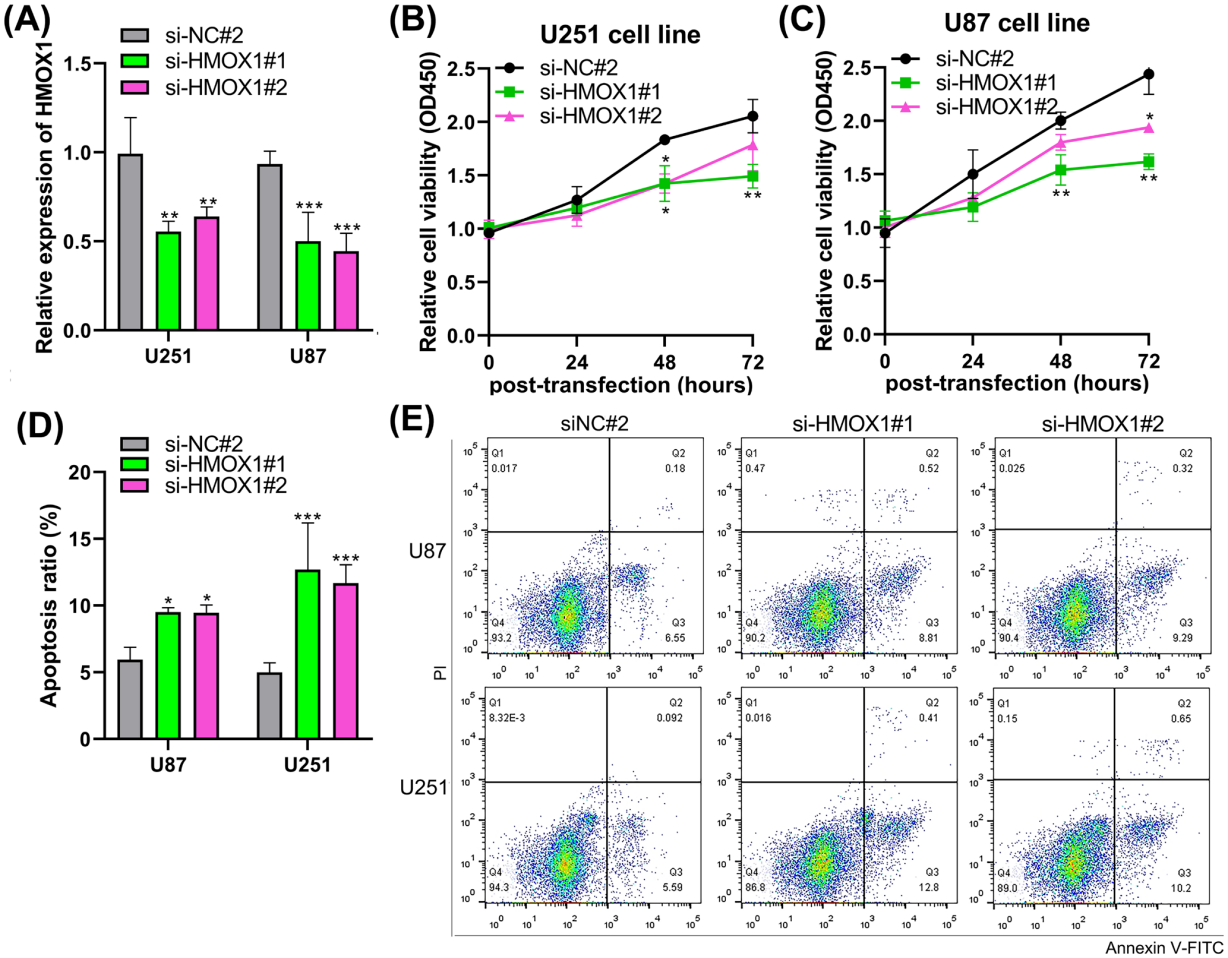
Downregulation of HMOX1 reduces cell viability and elevates apoptotic rates in glioma cells. (A) Expression levels of SPP1 in si‐HMOX1‐transfected cells, as determined by RT‐qPCR. (B, C) CCK‐8 assays were conducted to assess changes in cellular viability following HMOX1 downregulation. (D, E) Flow cytometric analysis demonstrates alterations in the apoptotic rate of glioma cells transfected with si‐HMOX1. *n* = 3. The data are presented as mean ± SD. *p < 0.05, **p < 0.01, ***p < 0.001.

To evaluate the potential role of SPP1/HMOX1 in promoting tumour progression in glioma, we examined whether silencing SPP1 and HMOX1 affects glioma cell viability and apoptosis. This analysis was achieved through transient transfection of siRNAs into U87MG and U251 cells. The downregulation of SPP1 was confirmed to be significant in the two glioma cell lines (*p* < 0.001, Figure 7C). SPP1 knockdown significantly inhibited the proliferative capacity of glioma cells in vitro, compared to the NC group (*p* < 0.05, *p* < 0.01, Figure 7D,E). In line with the cell viability results, a significant increase in the apoptotic ratio was observed in glioma cells after si‐SPP1 transfection, as shown by Annexin V/PI staining analysis (*p* < 0.05, *p* < 0.001, Figure 7F,G). Furthermore, CCK‐8 and flow cytometry also show the anti‐tumour effect of silencing HMOX1 in glioma (*p* < 0.05, *p* < 0.01, *p* < 0.001, Figure 8).

## 4 Discussion

The present study investigated the functional roles of SPP1 and HMOX1 in glioma and explored the downstream regulatory mechanism modulated by SPP1 and HMOX1. Our findings demonstrated that the elevated SPP1 and HMOX1 expression exhibited worse overall survival compared to those with lower expression levels of SPP1/HMOX1. The data from TCGA analysis showed that the expression levels of SPP1/HMOX1 were higher in WHO Grade IV (GBM) tissues than in the low‐grade glioma (LGG) group. Moreover, SPP1 and HMOX1 are directly associated with 20 key hub genes and play roles in regulating tumour progression in glioma. Notably, SPP1/HMOX1 and co‐expressed genes were related to activating the PI3K/AKT, JAK–STAT and syndecan 1 pathways in glioma, contributing to the tumour progression of glioma.

VSV encoding nonpathogenic negative‐stranded RNA has been evaluated as an inhibitory factor to decrease C6 glioblastoma cell growth and target chimeric antigen receptor (CAR)‐modified T cells [24, 25]. Lun et al. [26] altered the VSV virus by mutating the matrix (M) protein to enhance VSV's efficiency. The mutant VSV‐M51 prolonged survival of mice with glioma [26, 27]. Wollmann et al. [28] compared the oncolytic activity of ten recombinant VSV variants against GBM. The VSV‐M51 has almost no toxicity to glioma cells but has a rather good killing effect on tumour cells [28]. Together, these studies showed that stimulation through VSV‐M51 infection can be exploited to provide an advantage to inhibit glioma cell growth. Despite numerous advances in knowledge of VSV‐M51's anti‐tumour effects, many questions remain, particularly regarding the mechanisms of VSV‐M51 treatment and how these processes could be manipulated for therapeutic purposes. The current study is the first to demonstrate that SPP1 and HMOX1 downregulation in glioma cells infected with VSV‐M51 reveals a potential mechanism whereby VSV‐M51 infection modulates glioma.

Previous study has shown that the cell model expressing high SPP1 level exhibits cancer cell proliferation, migration, invasion and drug resistance to radiotherapy and chemotherapy [29, 30]. The high expression level of SPP1 is associated with increased macrophage infiltration and worse prognosis in glioma [31, 32]. Moreover, HMOX1 exhibits increased activity in cancer cells, resulting in the accumulation of tumour malignancy and the enhancement of apoptotic ability in ovarian cancer [33] and bladder cancer [34]. To explore the functional roles of SPP1 and HMOX1 in glioma, single‐cell analysis was performed based on their expression levels. GSEA analysis revealed the activation of cancer cell proliferation, EMT and tumour metastasis in the SPP1/HMOX1 overexpression group compared to the control group, indicating potential regulatory roles in glioma progression.

About 65%–90% of patients with LGG have IDH1/2 mutations, and IDH1/2 mutations have a significant correlation with improved prognosis compared with IDH‐wild type [35]. In recent years, studies have increasingly shown that treatment targeting IDH mutations plays extensive inhibitory roles in glioma tumour progression [1, 36]. Our findings highlight the importance of SPP1/HMOX1 overexpression in glioma, suggesting a potential correlation between SPP1/HMOX1 expression and IDH status in glioma. Moreover, our data reveal that elevated levels of SPP1/HMOX1 are observed across different glioma histological types, with higher expression noted in high‐grade glioma patients compared to those with low‐grade glioma. As high‐grade gliomas overexpressing SPP1/HMOX1 is associated with tumour progression and increased malignancy grade, genes co‐expressed with SPP1 and HMOX1 appear to be strongly associated with glioma development.

The PI3K/AKT pathway is a classical signal transduction pathway that plays a crucial regulatory role in glioma tumour progression [23]. Dysregulation of the PI3K/AKT pathway results in the activation of growth factors, which in turn promote glioma cell proliferation [37]. Previous studies have shown that temozolomide, a therapeutic agent targeting PI3K/AKT pathway emerges as a promising strategy for crossing the blood–brain barrier and minimising systemic toxicity [38]. A growing body of evidence suggests that the JAK–STAT pathway serves as a potential signalling module to activate different cellular responses and promote cancer progression and metastasis [39, 40]. The activation of JAK–STAT is commonly associated with the promotion of glioma pathobiology and oncogenesis [41]. Our study proposed, for the first time, that SPP1/HMOX1 contributeso the activation of the PI3K/AKT, JAK–STAT and syndecan 1 signalling pathways, thereby maintaining the motility and malignancy of glioma cells. Limitations of the current study include the lack of data regarding the regulatory mechanism of SPP1 and/or HMOX1 in glioma cell growth, as well as not providing in‐depth profiling for SPP1 and/or HMOX1 in different histologic grades of LGG or GBM. Further, in‐depth experimental validation studies are warranted to elucidate the mechanism of SPP1/HMOX1‐involved signalling pathways in glioma, and also to study their possible role and interrelation in cell migration and tumour metastasis.

In summary, the study identified that oncolytic virus VSV‐M51 treatment downregulated SPP1/HMOX1 expression in glioma cells. SPP1 and HMOX1 have the potential to be biomarkers for the diagnosis and prognosis of glioma. Our study elucidated an intricate association between SPP1/HMOX1 and multiple co‐expressed proteins in glioma, highlighting the potential role of SPP1/HMOX1 in regulating cell cycle and growth via the PI3K/AKT, JAK–STAT and syndecan 1 signalling pathways. Our results emphasised the importance of SPP1 and HMOX1 as novel targets for oncolytic virotherapy intervention, holding promise for glioma treatment.

## Author Contributions

**Chunze Cui:** conceptualization (equal), data curation (equal), formal analysis (equal), methodology (equal), validation (equal), visualization (equal), writing – original draft (equal), writing – review and editing (equal). **Chunyan Wu:** formal analysis (equal), software (equal), validation (equal), visualization (equal). **Shaoqi Zhang:** investigation (equal), methodology (equal), validation (equal), visualization (equal), writing – review and editing (equal). **Xiaofeng Yin:** conceptualization (equal), data curation (equal), formal analysis (equal), resources (equal), supervision (equal), validation (equal), visualization (equal), writing – original draft (equal), writing – review and editing (equal).

## Ethics Statement

The Ethics Committee of the Second Hospital of Shanxi Medical University evaluated and granted approval for this study.

## Consent

All participants provided written informed consent prior to their involvement in the study.

## Conflicts of Interest

The authors declare no conflicts of interest.

## Supporting information


**Figure S1.** Verification of prognostic values of SPP1 and HMOX1 in glioma. The expression levels of SPP1 and HMOX1 were associated with clinical outcomes within the TCGA LGG and GBM cohorts. The median value was used as the cutoff threshold to categorise glioma patients into low and high expression groups. Survival curves were generated using the Kaplan–Meier method.

## Data Availability

The data that support the findings of this study are available from the corresponding author upon reasonable request.

## References

[1] R. Chen , M. Smith‐Cohn , A. L. Cohen , and H. Colman , “Glioma Subclassifications and Their Clinical Significance,” Neurotherapeutics 14, no. 2 (2017): 284–297. 28281173 10.1007/s13311-017-0519-x PMC5398991

[2] S. Xu , L. Tang , X. Li , F. Fan , and Z. Liu , “Immunotherapy for Glioma: Current Management and Future Application,” Cancer Letters 476 (2020): 1–12. 32044356 10.1016/j.canlet.2020.02.002

[3] F. Yasinjan , Y. Xing , H. Geng , et al., “Immunotherapy: A Promising Approach for Glioma Treatment,” Frontiers in Immunology 14 (2023): 1255611. 37744349 10.3389/fimmu.2023.1255611 PMC10512462

[4] A. Pouyan , M. Ghorbanlo , M. Eslami , et al., “Glioblastoma Multiforme: Insights Into Pathogenesis, Key Signaling Pathways, and Therapeutic Strategies,” Molecular Cancer 24, no. 1 (2025): 58. 40011944 10.1186/s12943-025-02267-0 PMC11863469

[5] M. Weller , W. Wick , K. Aldape , et al., “Glioma,” Nature Reviews Disease Primers 1 (2015): 15017. 10.1038/nrdp.2015.17 27188790

[6] R. Ma , Z. Li , E. A. Chiocca , M. A. Caligiuri , and J. Yu , “The Emerging Field of Oncolytic Virus‐Based Cancer Immunotherapy,” Trends Cancer 9, no. 2 (2023): 122–139. 36402738 10.1016/j.trecan.2022.10.003 PMC9877109

[7] P. Muthukutty and S. Y. Yoo , “Oncolytic Virus Engineering and Utilizations: Cancer Immunotherapy Perspective,” Viruses 15, no. 8 (2023): 1645. 37631987 10.3390/v15081645 PMC10459766

[8] M. Mondal , J. Guo , P. He , and D. Zhou , “Recent Advances of Oncolytic Virus in Cancer Therapy,” Human Vaccines & Immunotherapeutics 16, no. 10 (2020): 2389–2402. 32078405 10.1080/21645515.2020.1723363 PMC7644205

[9] J. Raja , J. M. Ludwig , S. N. Gettinger , K. A. Schalper , and H. S. Kim , “Oncolytic Virus Immunotherapy: Future Prospects for Oncology,” Journal for Immunotherapy of Cancer 6, no. 1 (2018): 140. 30514385 10.1186/s40425-018-0458-z PMC6280382

[10] G. Tang , D. Wang , X. Zhao , Z. Feng , Q. Chen , and Y. Shen , “The Dilemma of HSV‐1 Oncolytic Virus Delivery: The Method Choice and Hurdles,” International Journal of Molecular Sciences 24, no. 4 (2023): 3681. 36835091 10.3390/ijms24043681 PMC9962028

[11] H. Fukuhara , Y. Ino , and T. Todo , “Oncolytic Virus Therapy: A New Era of Cancer Treatment at Dawn,” Cancer Science 107, no. 10 (2016): 1373–1379. 27486853 10.1111/cas.13027 PMC5084676

[12] H. Soliman , D. Hogue , H. Han , et al., “Oncolytic T‐VEC Virotherapy Plus Neoadjuvant Chemotherapy in Nonmetastatic Triple‐Negative Breast Cancer: A Phase 2 Trial,” Nature Medicine 29, no. 2 (2023): 450–457. 10.1038/s41591-023-02210-0 36759673

[13] K. J. Harrington , I. Puzanov , J. R. Hecht , et al., “Clinical Development of Talimogene Laherparepvec (T‐VEC): A Modified Herpes Simplex Virus Type‐1‐Derived Oncolytic Immunotherapy,” Expert Review of Anticancer Therapy 15, no. 12 (2015): 1389–1403. 26558498 10.1586/14737140.2015.1115725

[14] J. Rajwani , D. Vishnevskiy , M. Turk , et al., “VSV(∆M51) Drives CD8(+) T Cell‐Mediated Tumour Regression Through Infection of Both Cancer and Non‐Cancer Cells,” Nature Communications 15, no. 1 (2024): 9933. 10.1038/s41467-024-54111-6 PMC11567966 39548070

[15] P. Zeng , X. Zhang , T. Xiang , Z. Ling , C. Lin , and H. Diao , “Secreted Phosphoprotein 1 as a Potential Prognostic and Immunotherapy Biomarker in Multiple Human Cancers,” Bioengineered 13, no. 2 (2022): 3221–3239. 35067176 10.1080/21655979.2021.2020391 PMC8973783

[16] J. W. Eun , J. H. Yoon , H. R. Ahn , et al., “Cancer‐Associated Fibroblast‐Derived Secreted Phosphoprotein 1 Contributes to Resistance of Hepatocellular Carcinoma to Sorafenib and Lenvatinib,” Cancer Commun (Lond) 43, no. 4 (2023): 455–479. 36919193 10.1002/cac2.12414 PMC10091107

[17] P. Deepti , A. Pasha , D. V. Kumbhakar , et al., “Overexpression of Secreted Phosphoprotein 1 (SPP1) Predicts Poor Survival in HPV Positive Cervical Cancer,” Gene 824 (2022): 146381. 35271951 10.1016/j.gene.2022.146381

[18] P. Nallasamy , R. K. Nimmakayala , S. Karmakar , et al., “Pancreatic Tumor Microenvironment Factor Promotes Cancer Stemness via SPP1‐CD44 Axis,” Gastroenterology 161, no. 6 (2021): 1998–2013. 34418441 10.1053/j.gastro.2021.08.023 PMC10069715

[19] F. W. Hamilton , J. Somers , R. E. Mitchell , P. Ghazal , and N. J. Timpson , “HMOX1 Genetic Polymorphisms and Outcomes in Infectious Disease: A Systematic Review,” PLoS One 17, no. 5 (2022): e0267399. 35551540 10.1371/journal.pone.0267399 PMC9098073

[20] C. Zheng , B. Zhang , Y. Li , et al., “Donafenib and GSK‐J4 Synergistically Induce Ferroptosis in Liver Cancer by Upregulating HMOX1 Expression,” Advanced Science (Weinh) 10, no. 22 (2023): e2206798. 10.1002/advs.202206798 PMC10401117 37330650

[21] Z. Wang , W. Li , X. Wang , et al., “Isoliquiritigenin Induces HMOX1 and GPX4‐Mediated Ferroptosis in Gallbladder Cancer Cells,” Chinese Medical Journal 136, no. 18 (2023): 2210–2220. 37488674 10.1097/CM9.0000000000002675 PMC10508381

[22] M. Ni , J. Zhou , Z. Zhu , et al., “Shikonin and Cisplatin Synergistically Overcome Cisplatin Resistance of Ovarian Cancer by Inducing Ferroptosis via Upregulation of HMOX1 to Promote Fe(2+) Accumulation,” Phytomedicine 112 (2023): 154701. 36773431 10.1016/j.phymed.2023.154701

[23] E. Mohamed , A. Kumar , Y. Zhang , et al., “PI3K/AKT/mTOR Signaling Pathway Activity in IDH‐Mutant Diffuse Glioma and Clinical Implications,” Neuro‐Oncology 24, no. 9 (2022): 1471–1481. 35287169 10.1093/neuonc/noac064 PMC9435510

[24] L. Evgin , T. Kottke , J. Tonne , et al., “Oncolytic Virus‐Mediated Expansion of Dual‐Specific CAR T Cells Improves Efficacy Against Solid Tumors in Mice,” Science Translational Medicine 14, no. 640 (2022): eabn2231. 35417192 10.1126/scitranslmed.abn2231 PMC9297825

[25] S. Balachandran and G. N. Barber , “Vesicular Stomatitis Virus (VSV) Therapy of Tumors,” IUBMB Life 50, no. 2 (2000): 135–138. 11185959 10.1080/713803696

[26] X. Lun , D. L. Senger , T. Alain , et al., “Effects of Intravenously Administered Recombinant Vesicular Stomatitis Virus (VSV(deltaM51)) on Multifocal and Invasive Gliomas,” Journal of the National Cancer Institute 98, no. 21 (2006): 1546–1557. 17077357 10.1093/jnci/djj413

[27] B. Jiang , D. Huang , W. He , et al., “Inhibition of Glioma Using a Novel Non‐Neurotoxic Vesicular Stomatitis Virus,” Neurosurgical Focus 50, no. 2 (2021): E9. 10.3171/2020.11.FOCUS20839 33524950

[28] G. Wollmann , V. Rogulin , I. Simon , J. K. Rose , and A. N. van den Pol , “Some Attenuated Variants of Vesicular Stomatitis Virus Show Enhanced Oncolytic Activity Against Human Glioblastoma Cells Relative to Normal Brain Cells,” Journal of Virology 84, no. 3 (2010): 1563–1573. 19906910 10.1128/JVI.02040-09 PMC2812324

[29] E. Matsubara , H. Yano , C. Pan , et al., “The Significance of SPP1 in Lung Cancers and Its Impact as a Marker for Protumor Tumor‐Associated Macrophages,” Cancers (Basel) 15, no. 8 (2023): 2250. 37190178 10.3390/cancers15082250 PMC10136569

[30] G. Deng , F. Zeng , J. Su , et al., “BET Inhibitor Suppresses Melanoma Progression via the Noncanonical NF‐κB/SPP1 Pathway,” Theranostics 10, no. 25 (2020): 11428–11443. 33052224 10.7150/thno.47432 PMC7546000

[31] C. He , L. Sheng , D. Pan , et al., “Single‐Cell Transcriptomic Analysis Revealed a Critical Role of SPP1/CD44‐Mediated Crosstalk Between Macrophages and Cancer Cells in Glioma,” Frontiers in Cell and Development Biology 9 (2021): 779319. 10.3389/fcell.2021.779319 PMC8602110 34805184

[32] W. Tang , C. W. S. Lo , W. Ma , A. T. W. Chu , A. H. Y. Tong , and B. H. Y. Chung , “Revealing the Role of SPP1(+) Macrophages in Glioma Prognosis and Therapeutic Targeting by Investigating Tumor‐Associated Macrophage Landscape in Grade 2 and 3 Gliomas,” Cell & Bioscience 14, no. 1 (2024): 37. 38515213 10.1186/s13578-024-01218-4 PMC10956315

[33] J. Huang and R. Tan , “HMOX1: A Pivotal Regulator of Prognosis and Immune Dynamics in Ovarian Cancer,” BMC Womens Health 24, no. 1 (2024): 476. 39210460 10.1186/s12905-024-03309-3 PMC11363456

[34] M. S. Yim , Y. S. Ha , I. Y. Kim , S. J. Yun , Y. H. Choi , and W. J. Kim , “HMOX1 Is an Important Prognostic Indicator of Nonmuscle Invasive Bladder Cancer Recurrence and Progression,” Journal of Urology 185, no. 2 (2011): 701–705. 21168882 10.1016/j.juro.2010.09.081

[35] J. J. Miller , “Targeting IDH‐Mutant Glioma,” Neurotherapeutics 19, no. 6 (2022): 1724–1732. 35476295 10.1007/s13311-022-01238-3 PMC9723039

[36] R. V. Lukas and C. Horbinski , “Glioma Response to IDH Inhibition: Real‐World Experience,” Clinical Cancer Research 29, no. 23 (2023): 4709–4710. 37738033 10.1158/1078-0432.CCR-23-2164 PMC10840794

[37] E. Obrador , P. Moreno‐Murciano , M. Oriol‐Caballo , et al., “Glioblastoma Therapy: Past, Present and Future,” International Journal of Molecular Sciences 25, no. 5 (2024): 2529. 38473776 10.3390/ijms25052529 PMC10931797

[38] D. Wang , Z. Wang , X. Dai , L. Zhang , and M. Li , “Apigenin and Temozolomide Synergistically Inhibit Glioma Growth Through the PI3K/AKT Pathway,” Cancer Biotherapy and Radiopharmaceuticals 39, no. 2 (2024): 125–132. 33471569 10.1089/cbr.2020.4283

[39] A. Valle‐Mendiola , A. Gutiérrez‐Hoya , and I. Soto‐Cruz , “JAK/STAT Signaling and Cervical Cancer: From the Cell Surface to the Nucleus,” Genes (Basel) 14, no. 6 (2023): 1141. 37372319 10.3390/genes14061141 PMC10298571

[40] F. Shao , X. Pang , and G. H. Baeg , “Targeting the JAK/STAT Signaling Pathway for Breast Cancer,” Current Medicinal Chemistry 28, no. 25 (2021): 5137–5151. 33290193 10.2174/0929867328666201207202012

[41] K. Swiatek‐Machado and B. Kaminska , “STAT Signaling in Glioma Cells,” Advances in Experimental Medicine and Biology 1202 (2020): 203–222. 32034715 10.1007/978-3-030-30651-9_10

