# Supplementary figures and images for: Impact of SPP1 and HMOX1 Genes in Glioma: Correlations With Oncolytic Virus Infection, Adverse Prognosis and Increased Cell Proliferation
Source: J Cell Mol Med. 2025 Jun 11;29(11):e70651. doi: 10.1111/jcmm.70651 (PMC12152367; doi:10.1111/jcmm.70651)

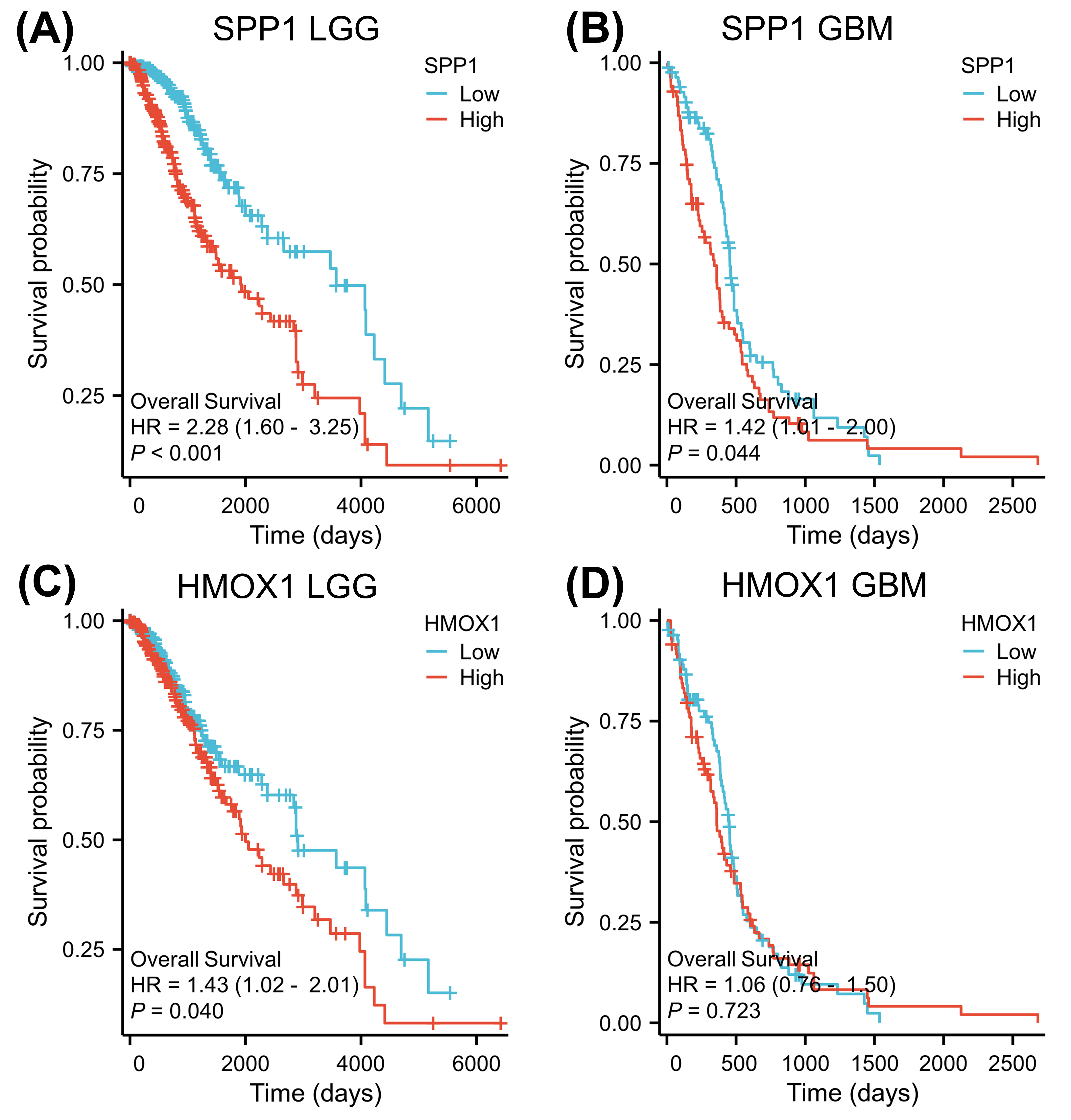

Supplement: Supplementary file 1 — Figure S1. Verification of prognostic values of SPP1 and HMOX1 in glioma. The expression levels of SPP1 and HMOX1 were associated with clinical outcomes within the TCGA LGG and GBM cohorts. The median value was used as the cutoff threshold to categorise glioma patients into low and high expression groups. Survival curves were generated using the Kaplan–Meier method. [file JCMM-29-e70651-s001.tif]
